# Serum procalcitonin level correlates with endotoxin activity in patients with septic shock

**DOI:** 10.1186/cc14137

**Published:** 2015-03-16

**Authors:** B Adamik, J Smiechowicz, D Jakubczyk, B Adamiczka-Ciszewicz, T Kaiser, A Kübler

**Affiliations:** 1 Medical University, Wroclaw, Poland

## Introduction

Endotoxin, a key component on the outer membrane of Gram-negative bacteria, is considered to be the most important toxin involved in the development of septic shock, and procalcitonin (PCT) serum concentration has been strongly associated with the severity of sepsis. The effect of elevated endotoxin activity (EA) on PCT serum level, organ function and mortality of patients with septic shock was evaluated on the day of admission to the ICU.

## Methods

ICU patients with diagnosis of septic shock were consecutively added to the study group within the first 24 hours. Serum PCT level and whole blood EA was immediately measured in all patients on admission (*n *= 157). Endotoxemia was defined as EA >0.4 EAU.

## Results

Endotoxemia was present in 61% of patients (group 1, *n *= 95, age 66 (57 to 75)), and in 39% of patients EA was low (group 2, *n *= 62, age 63 (55 to 76)). Median EA was 0.57 EAU (0.46 to 0.67) in group 1 and 0.27 EAU (0.17 to 0.36) in group 2 (*P *< 0.001). The PCT level was six times higher in group 1 than in group 2 (19.6 ng/ml vs. 3.1 ng/ml, *P *< 0.001) and was correlated with EA (*P *< 0.001, *R *= 0.5). Median APACHE II score was 23 points (16 to 29) in group 1 and 19 (16 to 25) in group 2; but observed difference was not significant. The severity of clinical status estimated by SOFA score was similar in both groups (10 (7 to 13) in group 1 and 11 (8 to 12) in group 2; NS). Forty-six percent of patients in group 1 and 27% in group 2 required renal replacement therapy (*P *= 0.01). ICU mortality of patients was 41%. The mortality rate was higher in group 1, compared with group 2, and Kaplan-Meier survival analysis of time to death showed statistical significance between the two groups (*P *= 0.001, log-rank test). A Gram-negative pathogen as the primary source of infection was identified in 64% of patients in group 1 and in 44% in group 2 (*P *= 0.004); bacteremia was detected in 26% of cases in group 1 and in 12% in group 2 (*P *= 0.02).

## Conclusion

Septic shock with endotoxemia was associated with biochemical and clinical consequences including a higher PCT level, higher frequency of bacteremia, kidney failure, and death.

